
JOURNAL INFORMATION
==============================
NLM Title Abbreviation: QJM
Journal ID: QJM
NLM Journal ID: 9438285
Journal ID: qjmedj

QJM: An International Journal of Medicine

ISSN: 1460-2725
EISSN: 1460-2393

ARTICLE INFORMATION
==============================
PMCID: PMC8690360
PMID: 34865168
DOI: 10.1093/qjmed/hcab307
Article ID: hcab307
Article version: 1
Subjects: Commentary, AcademicSubjects/MED00010

Why do breakthrough COVID-19 infections occur in the vaccinated?

https://orcid.org/0000-0001-5702-9929 Maclean Patrick MBBS BMedSc 1
Mentzer Alexander J BSc MSc MRCP DPhil 1
Lambe Teresa PhD OBE 2
Knight Julian 1
1 Wellcome Centre for Human Genetics, University of Oxford , Oxford, UK
2 Oxford Vaccine Group, Department of Paediatrics, University of Oxford, Oxford, UK; Chinese Academy of Medical Science (CAMS) Oxford Institute, University of Oxford , Oxford, UK
Co-corresponding author— patrick.maclean@merton.ox.ac.uk, julian@well.ox.ac.uk
Electronic publication date: 2021 Dec 4
Electronic Location ID: hcab307
Received 2021 Nov 22; Revised 2021 Nov 25
Copyright: © The Author(s) 2021. Published by Oxford University Press on behalf of the Association of Physicians.
Copyright year: 2021
License: This is an Open Access article distributed under the terms of the Creative Commons Attribution License (https://creativecommons.org/licenses/by/4.0/), which permits unrestricted reuse, distribution, and reproduction in any medium, provided the original work is properly cited.
License URL: https://creativecommons.org/licenses/by/4.0/

Page count: 5
edited-state: accepted-manuscript
article-lifecycle: PAP

==============================

### Why do breakthrough COVID-19 infections occur in the vaccinated?

List of authors:

**1.**
Dr Patrick Maclean MBBS BMedSc
Co-corresponding author - patrick.maclean@merton.ox.ac.uk
DPhil student
Wellcome Centre for Human Genetics, University of Oxford, Oxford, UK.
ORCID 0000-0001-5702-9929

2.
Dr Alexander J. Mentzer BSc MSc MRCP DPhil
Group Leader
Wellcome Centre for Human Genetics, University of Oxford, Oxford, UK.

3.
Professor Teresa Lambe PhD OBE
Oxford Vaccine Group, Department of Paediatrics, University of Oxford, Oxford, UK; Chinese Academy of Medical Science (CAMS) Oxford Institute, University of Oxford, Oxford, UK.

4.
Prof Julian Knight
Co-corresponding author – julian@well.ox.ac.uk
Professor of Genomic Medicine
Deputy Director, Wellcome Centre for Human Genetics, University of Oxford, Oxford, UK.
Honorary Consultant Physician

© The Author(s) 2021. Published by Oxford University Press on behalf of the Association of Physicians.
This is an Open Access article distributed under the terms of the Creative Commons Attribution License (http://creativecommons.org/licenses/by/4.0/), which permits unrestricted reuse, distribution, and reproduction in any medium, provided the original work is properly cited.

More than 7 billion vaccinations against SARS-CoV-2 have now been administered.[1] Clinical trials and national epidemiological studies have shown that a variety of vaccines are effective in reducing cases, hospital admissions and deaths from COVID-19 in the months after vaccination. However despite effective vaccine campaigns in many countries, small but increasing numbers of breakthrough infections are being reported in fully vaccinated people.

**Clinical presentation of breakthrough COVID-19**

The majority of breakthrough infections reported to date are mild, although early studies have been biased towards cohorts of healthcare workers and the comparatively healthy patients included in clinical trials. An Israeli study, published in July, reported 39 mild or asymptomatic breakthrough infections among 1497 healthcare workers who were a median of 39 days from their second dose of the BNT162b2 vaccine.[2]

Data from the UK ZOE COVID Symptom Study show a similar pattern. In 2370 self-reported breakthrough cases drawn from 1.2 million people who received the ChAdOx nCov-19 or BNT162b2 vaccine with an extended dosing interval, the likelihood of a case being asymptomatic was higher in the vaccinated.[3] All COVID-19 symptoms were less frequently reported and of shorter duration in the vaccinated cases than in the unvaccinated, but when vaccinated cases were symptomatic they were most likely to report headache, rhinorrhea, sneeze, sore throat and/or anosmia. Vaccinated cases were also half as likely to report experiencing symptoms for 28 days or longer (odds ratio 0.51, 95% CI 0.32 – 0.82). This evidence suggests that the ‘core symptoms’ of fever and continuous cough used in UK public health guidance may need to be updated to match the symptoms seen in breakthrough COVID-19.

Population-wide datasets show that breakthrough infections can be more severe in some people. A UK study using a national administrative dataset applied a clinical risk scoring algorithm to predict hospitalisation and death due to COVID-19 after vaccination.[4] Vaccination universally reduced the likelihood of severe outcomes without altering the predictors of severe disease or death, with the strongest predictors of COVID-19 mortality in the United Kingdom remaining older age, socioeconomic deprivation, male sex and Indian and Pakistani ethnic origin. Condition-specific hazard ratios were highest for conditions which have been associated with reduced immune responsiveness after vaccination, including recent chemotherapy, HIV/AIDS infection and recent bone marrow transplantation or history of solid organ transplantation.

This patient profile was mirrored in an Israeli cohort of 152 patients hospitalised with breakthrough infections after receiving the BNT162b2 vaccine, albeit using the three week dosing interval employed in Israel. The majority of these patients were elderly and multiply comorbid, with 40% immunocompromised and only 4% free of all comorbidities.[5]

**Waning vaccine effectiveness over time**

Emerging evidence shows that the risk of severe breakthrough infection increases in the months following vaccination. A test-negative case-control study conducted by Public Health England, published as a preprint and containing data up to September 2021, showed that vaccine effectiveness against symptomatic disease begins to decline around 10 weeks from vaccination, with a smaller decline in effectiveness against hospitalisation and death noted from 15 weeks.[6] While effectiveness against hospitalisation remained 77% (95% CI 70.3-82.3%) and 92.7% (95% CI 90.3-94.6%) for the ChAdOx nCov-19 and BNT162b2 vaccines respectively, the study showed that effectiveness declined further in people who were over 65 or clinically vulnerable.

**Correlates of protection**

Data from the randomised efficacy trials of the ChAdOx1 nCoV-19 vaccine showed that the level of neutralising antibodies directed against the Spike protein receptor binding domain, measured 28 days after the second dose, has some utility in predicting protection against symptomatic infection, though no cut-off delineating absolute protection could be defined.[7]

While neutralising antibodies are not a perfect correlate of protection, some inferences can be drawn. Measurements of antibody levels from the ONS National COVID-19 Infection Survey have identified a ‘low antibody responder’ group which overlaps significantly with the groups shown to be at risk of breakthrough infection, being enriched for males, people aged 75 or older and those with chronic comorbidities and immunosuppression, as well as possibly those with rare genetic variants. [8] Detailed immunotyping of these patients, and identification of those who remain protected despite recording low antibody titres, will help to elucidate how mechanisms of immunity beyond serum antibody titres contribute to protection after vaccination.

**Booster vaccinations**

Antibody levels are not static and are expected to wane, with the kinetics of the waning process varying across different vaccines and dosing schedules. A third dose of the ChAdOx1 nCoV-19 vaccine given 6-9 months after the primary course has been shown to restore neutralising antibody levels to a similar range to that seen after the primary course.[9] Headline, non peer-reviewed results of a large phase III randomised, controlled trial testing a third dose of the BNT162b2 vaccine report that efficacy against symptomatic disease is restored for at least the first 2.5 months after the third dose, however neither the longevity of this restored protection against symptomatic disease or measures of protection against severe disease or death have yet been reported. While booster campaigns have now commenced in many countries, the extent to which people who responded poorly to the primary course will respond either clinically or immunologically to another dose remains unknown.

**Conclusions**

As breakthrough cases accrue around the world it is important for clinicians and scientists to know which people are at the highest risk of severe breakthrough infections. Longer followup of vaccinated individuals will provide more information on the durability of the immune response to vaccination and identify genetic and environment factors associated with varying immune responsiveness. The progress of booster programs should be closely monitored to investigate their effectiveness in people with low immune responses to their primary course, and to test alternate dosing strategies. Human challenge trials, currently underway in the UK, will help to investigate the immunological response to reinfection at a level of granularity unmatched by population studies. Together these data should inform expectations about the disease spectrum of COVID-19 in the endemic stage, and guide the design of a long term vaccination strategy for the world.

1. Dong E, Du H, Gardner L. An interactive web-based dashboard to track COVID-19 in real time. Lancet Infect Dis. 2020 May 1;20(5):533–4.

2. Bergwerk M, Gonen T, Lustig Y, Amit S, Lipsitch M, Cohen C, et al. Covid-19 Breakthrough Infections in Vaccinated Health Care Workers. N Engl J Med. 2021 Jul 28;385(16):1474–84.

3. Antonelli M, Penfold RS, Merino J, Sudre CH, Molteni E, Berry S, et al. Risk factors and disease profile of post-vaccination SARS-CoV-2 infection in UK users of the COVID Symptom Study app: a prospective, community-based, nested, case-control study. Lancet Infect Dis. Published online 1 Sep 2021.

4. Hippisley-Cox J, Coupland CA, Mehta N, Keogh RH, Diaz-Ordaz K, Khunti K, et al. Risk prediction of covid-19 related death and hospital admission in adults after covid-19 vaccination: national prospective cohort study. BMJ. 2021 Sep 17;374:n2244.

5. Brosh-Nissimov T, Orenbuch-Harroch E, Chowers M, Elbaz M, Nesher L, Stein M, et al. BNT162b2 vaccine breakthrough: clinical characteristics of 152 fully vaccinated hospitalized COVID-19 patients in Israel. Clin Microbiol Infect. Published online 7 Jul 2021.

6. Andrews N, Tessier E, Stowe J, Gower C, Kirsebom F, Simmons R, et al. Vaccine effectiveness and duration of protection of Comirnaty, Vaxzevria and Spikevax against mild and severe COVID-19 in the UK. Medrxiv. Published online as preprint 6 Oct 2021.

7. Feng S, Phillips DJ, White T, Sayal H, Aley PK, Bibi S, et al. Correlates of protection against symptomatic and asymptomatic SARS-CoV-2 infection. Nat Med. Published online 29 Sep 2021.

8. Wei J, Stoesser N, Matthews PC, Ayoubkhani D, Studley R, Bell I, et al. Antibody responses to SARS-CoV-2 vaccines in 45,965 adults from the general population of the United Kingdom. Nat Microbiol. 2021 Sep;6(9):1140–9.

9. Flaxman A, Marchevsky NG, Jenkin D, Aboagye J, Aley PK, Angus B, et al. Reactogenicity and immunogenicity after a late second dose or a third dose of ChAdOx1 nCoV-19 in the UK: a substudy of two randomised controlled trials (COV001 and COV002). The Lancet. 2021 Sep 11;398(10304):981–90.

10. Pfizer, BioNTech. Pfizer and BioNTech announce phase 3 trial data showing high efficacy of a booster dose of their COVID-19 vaccine [Internet]. pfizer.com. Published online 21 Oct 2021. Available from: https://www.pfizer.com/news/press-release/press-release-detail/pfizer-and-biontech-announce-phase-3-trial-data-showing
